# Incident and recurrent herpes zoster for first-line bDMARD and tsDMARD users in seropositive rheumatoid arthritis patients: a nationwide cohort study

**DOI:** 10.1186/s13075-022-02871-1

**Published:** 2022-07-28

**Authors:** Seogsong Jeong, Seulggie Choi, Sang Min Park, Jinseok Kim, Byeongzu Ghang, Eun Young Lee

**Affiliations:** 1 Department of Biomedical Sciences, Seoul National University College of Medicine, Seoul, South Korea; 2 Department of Biomedical Informatics, CHA University School of Medicine, CHA University, Seongnam, South Korea; 3 Department of Internal Medicine, Seoul National University Hospital, Seoul, South Korea; 4 Department of Family Medicine, Seoul National University Hospital, Seoul, South Korea; 5 Division of Rheumatology, Department of Internal Medicine, Jeju National University School of Medicine, Jeju National University Hospital, Jeju, South Korea; 6 Division of Rheumatology, Department of Internal Medicine, Seoul National University College of Medicine, 101 Daehak-ro, Jongno-gu, Seoul, South Korea; 7 Integrated Major in Innovative Medical Science, Seoul National University Graduate School, Seoul, South Korea

**Keywords:** Herpes zoster, Rheumatoid arthritis, Biologic disease-modifying antirheumatic drugs, Targeted synthetic disease-modifying antirheumatic drugs

## Abstract

**Background:**

There is limited information regarding disease-modifying antirheumatic drug (DMARD)-dependent risks of overall, incident, and recurrent herpes zoster (HZ) during first-line biologic DMARD (bDMARD) or targeted synthetic DMARD (tsDMARD) treatment among patients with seropositive rheumatoid arthritis (RA) in terms of HZ risk.

**Methods:**

A total of 11,720 patients with seropositive RA who were prescribed bDMARD or tofacitinib between January 2011 and January 2019 from the Korean Health Insurance Review & Assessment Service database were studied. A multivariate Cox proportional hazards regression model was adopted to evaluate the adjusted hazard ratio (aHR) with 95% confidence interval (CI) for the risk of HZ dependent on the choice of first-line bDMARDs or tsDMARD, including etanercept, infliximab, adalimumab, golimumab, tocilizumab, rituximab, tofacitinib, and abatacept.

**Results:**

During the 34,702 person-years of follow-up, 1686 cases (14.4%) of HZ were identified, including 1372 (11.7%) incident and 314 (2.7%) recurrent HZs. Compared with that of the abatacept group, tofacitinib increased the overall risk (aHR, 2.46; 95% CI, 1.61–3.76; *P*<0.001), incidence (aHR, 1.99; 95% CI, 1.18–3.37; *P*=0.011), and recurrence (aHR, 3.69; 95% CI, 1.77–7.69; *P*<0.001) of HZ. Infliximab (aHR, 1.36; 95% CI, 1.06–1.74; *P*=0.017) and adalimumab (aHR, 1.29; 95% CI, 1.02–1.64; *P*=0.032) also increased the overall HZ risk. Moreover, a history of HZ was found to be an independent risk factor for HZ (aHR, 1.54; 95% CI, 1.33–1.78; *P*<0.001).

**Conclusions:**

HZ risk is significantly increased in RA patients with a history of HZ after the initiation of bDMARDs or tsDMARD. The risk of incident and recurrent HZ was higher after tofacitinib treatment in patients with RA than that after treatment with bDMARDs. Individualized characteristics and history of HZ should be considered when selecting bDMARDs or tsDMARD for RA patients considering HZ risks.

**Supplementary Information:**

The online version contains supplementary material available at 10.1186/s13075-022-02871-1.

## Introduction

Rheumatoid arthritis (RA) is a systemic autoimmune disease characterized by symmetrical inflammatory polyarthritis that commonly begins in small joints, with approximately 20 million prevalent cases worldwide [1]. Achieving sustained remission using conventional synthetic disease-modifying antirheumatic drugs (DMARDs) is considered the standard of care [2]. However, approximately 30–50% of patients respond inadequately to traditional DMARDs, and biologic DMARDs (bDMARDs) and targeted synthetic DMARDs (tsDMARD) are considered if the response is inadequate after 2–6 months of methotrexate therapy [3, 4].

The long-term clinical consequences of RA are more likely to result in diminished health-related quality of life compared to that of the general population [5]. The risk of serious conditions such as infection, cardiovascular disease, respiratory disease, osteoporosis, and cancer is increased in patients with RA, which is suggested to be associated with autoimmunity, chronic inflammation, and immunosuppression [6–8]. A number of previous studies revealed that patients with RA have a significantly increased risk of herpes zoster (HZ) and suggested a potential association between DMARDs and HZ [9, 10]. The association between tumor necrosis factor (TNF) inhibitors and non-TNF inhibitors with HZ risk remains controversial, and a pooled analysis reported that non-TNF blockers, but not TNF blockers, were associated with higher HZ risk [11–13]. In addition, Curtis et al. [14] found that tofacitinib approximately doubled the rate of HZ compared with that of other biologics.

The incidence and recurrence of HZ are quite common in patients with RA in real-world clinical settings. However, there is limited evidence regarding bDMARD-dependent HZ risk among patients with a history of HZ prior to bDMARD use. In addition, newly diagnosed HZ was found to be associated with a higher incidence of cardio-cerebrovascular disease than those without HZ, potentially due to inflammation and subsequent thrombosis [15, 16]. Therefore, concerns regarding bDMARD-dependent HZ risk have become important in real-world clinical settings. Herein, we explored the associations of first-line bDMARD with overall, incident, and recurrent HZ risks among patients with RA during the first bDMARD treatment using the Korean Health Insurance Review & Assessment Service (HIRA) database.

## Patients and methods

### Study population

All Korean citizens are enrolled in a mandatory health insurance service managed by the Korean National Health Insurance Service (NHIS) that covers almost all forms of health services [17]. Healthcare providers in Korea send patient data to the HIRA regarding all health services covered by the insurance for reimbursements [18]. The HIRA database includes sociodemographic characteristics, such as age, sex, body mass index, area of residence, insurance premium, and hospital visit information, such as diagnosis codes and pharmaceutical prescriptions, and provides a part of the database for research purposes. Several studies have been conducted using the HIRA database, and its validity has been presented in previous studies [19].

**Fig. 1:**
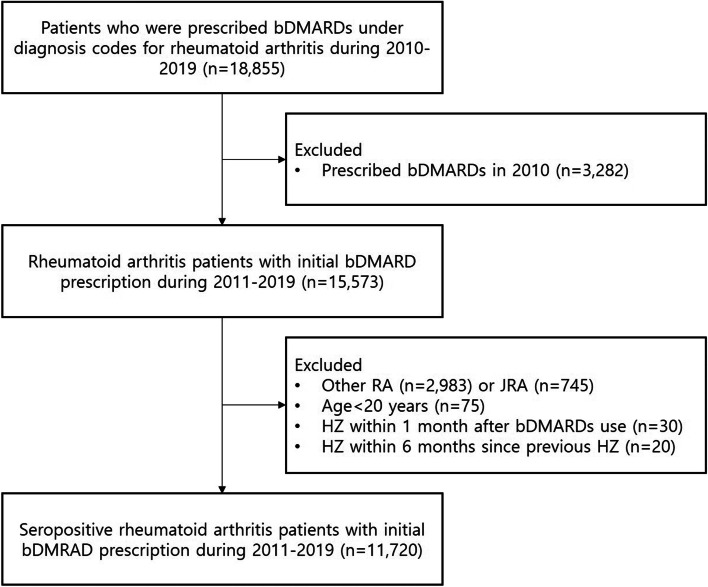
Patient inclusion flowchart. Participants who were prescribed with the first-line bDMARDs during 2011 and 2019 were included after excluding other rheumatoid arthritis or juvenile rheumatoid arthritis, development of herpes zoster within 1 month after bDMARDs, and recurrent herpes zoster that occurred within 6 months since the date of the previous herpes zoster infection

A total of 18,855 patients with seropositive RA who were prescribed bDMARDs or tofacitinib between January 2010 and January 2019 were identified. In the present study, TNF inhibitors (etanercept, infliximab, adalimumab, and golimumab), non-TNF inhibitors (tocilizumab [interleukin-6 receptor blocker], rituximab [CD20 inhibitor], abatacept [T-cell costimulation inhibitor]), and tofacitinib (Janus kinase inhibitor) were considered bDMARDs and tsDMARD. First, bDMARD and tsDMARD use were operationally defined by excluding those who were prescribed bDMARDs and tsDMARD in 2010 (*n* = 3282) to limit the study population to new users. In addition, patients with non-seropositive RA (*n* = 2983) or juvenile RA (*n* = 745), age <20 years (*n* = 75), HZ within 1 month of bDMARD or tsDMARD use (*n* = 30), and HZ within 6 months of previous HZ (*n* = 20) were excluded. To exclude patients with non-seropositive and juvenile RA, these conditions were evaluated from January 2010 to the date of bDMARD or tsDMARD initiation. HZ within 6 months after the previous HZ was defined as those with prior HZ before the initiation of bDMARD or tsDMARD treatment who had HZ within 6 months since the date of the previous HZ after the initiation of bDMARD or tsDMARD treatment. Finally, the analytic cohort included 11,720 patients with seropositive RA, which was defined using the International Classification of Diseases Tenth Revision code of M05 as adopted from previous studies, who were prescribed an initial bDMARD or tofacitinib between January 2011 and January 2019 (Fig. 1) [20, 21]. An additional study design is shown in Supplementary Fig. 1. Follow-up investigations were performed for all patients until drug failure, development of HZ, or January 31, 2019, whichever occurred earlier. The prescription of bDMARDs or tsDMARD was based on drug codes in the HIRA database. Drug failure was defined as the prescription of different bDMARDs and tsDMARD from the original bDMARDs or tsDMARD, in accordance with a previous study [21]. Patients with longer than 1 year of refill period from the date of the last drug prescription were censored at the date of the last drug prescription. The drug failure-free survival was calculated for each individual to define person-days of bDMARDs or tsDMARD treatment for the follow-up investigation. This study was approved by the Institutional Review Board of Seoul National University Hospital (2002-057-1100). The requirement for informed consent was waived as the HIRA database was anonymized with strict confidentiality guidelines.

### Outcomes

HZ was considered to be present according to the International Classification of Diseases tenth revision diagnosis code for HZ, which is B02, during the first-line prescription of bDMARDs or tsDMARD [22]. History of HZ was defined as individuals who had HZ prior to the initial bDMARD or tsDMARD treatment. Recurrent HZ was defined as the development of HZ after initiation of bDMARD or tsDMARD treatment in patients with a history of HZ that developed before the initiation of bDMARD or tsDMARD treatment.

### Key variables

The following variables were considered for adjustments: age (continuous; year), sex (categorical; men and women), number of conventional synthetic DMARD types at initiation of bDMARDs or tsDMARD (continuous), Charlson comorbidity index (CCI; continuous), enrollment year (categorical; 2011 to 2019), glucocorticoid use within 6 months after the date of initial bDMARD and tofacitinib use (continuous, mg/day), history of HZ (categorical; yes and no), the period between the date of the previous HZ and the date of initial bDMARD and tofacitinib use (continuous, year), and glucocorticoid use (continuous, mg/day). Azathioprine, cyclosporine, cyclophosphamide, hydroxychloroquine sulfate, leflunomide, methotrexate, and sulfasalazine were considered conventional synthetic DMARD (number of conventional synthetic DMARDs within 1 year prior to the bDMARD or tsDMARD prescription). For stratified analyses, the following variables were selected: sex (men and women), age (≥65 years and <65 years), number of conventional synthetic DMARD types (1–2 and >2), CCI (≤2 and >2), and glucocorticoid use within 6 months after the date of initial bDMARD or tsDMARD use (≥5 mg/day and <5 mg/day).

### Statistical analysis

Analysis of variance and chi-square test were applied for continuous and categorical variables for the statistical evaluation of differences. A multivariate Cox proportional hazards regression model was adopted to calculate the adjusted hazard ratio (aHR) with 95% confidence interval (CI) for the evaluation of the association between initial bDMARDs or tsDMARD and incident or recurrent HZ. For data interpretation, abatacept was used as the reference, according to a previous study [14]. Kaplan–Meier estimation was carried out to describe the initial and recurrent HZ-free survival. Incidence was calculated as the number of event/1000 person-years of follow-up. Sensitivity analyses were performed to support the main findings by excluding HZ cases that occurred within 1, 3, and 5 years after the follow-up investigation. Subgroup analyses were performed after stratifying patients according to sex, age, CCI, and glucocorticoid use to identify potential interactions. Patients with missing data, such as variables for adjustment, were excluded from the study. Statistical significance was set at *P* < 0.05. All data collection, mining, and statistical analyses were performed using the SAS Enterprise Guide 7.1 (SAS Institute, USA).

## Results

### Patient characteristic

All patients were stratified into the etanercept (*n* = 2680), infliximab (*n* = 1315), adalimumab (*n* = 3229), golimumab (*n* = 1197), tocilizumab (*n* = 1378), rituximab (*n* = 67), tofacitinib (*n* = 701), and abatacept (*n* = 1153) groups. There were 2152 men (18.4%) and 9568 women (81.6%), with a median age of 54.8 years (Table 1). Patients who received abatacept were relatively older (median age, 60 years). A total of 1790 patients (15.3%) were prescribed ≤1 DMARD. Within 6 months after the initial bDMARD or tsDMARD use, patients had a median of 4.5 mg/day (interquartile range [IQR], 2.8–6.2) of glucocorticoid use. In addition, 5080 patients (43.3%) and 4125 patients (35.2%) had a CCI of 1–2 and 3–4, respectively. The median follow-up period was 2.7 years (IQR, 0.8–5.7).

**Table 1:** Descriptive characteristics of rheumatoid arthritis patients during first bDMARD or tsDMARD use

	Total	TNFα inhibitor				Non-TNFα inhibitor			
		Etanercept	Infliximab	Adalimumab	Golimumab	Tocilizumab	Rituximab	Tofacitinib	Abatacept
Number of participants, *N* (%)	11,720	2680 (22.9)	1315 (11.2)	3229 (27.6)	1197 (10.2)	1378 (11.8)	67 (0.6)	701 (6.0)	1153 (9.8)
Age, years, median (IQR)	56 (47–64)	55 (45–64)	56 (48–63)	54 (44–62)	55 (46–64)	57 (49–65)	60 (50–67)	56 (47–63)	60 (52–68)
Sex, *N* (%)									
Men	2152 (18.4)	518 (19.3)	217 (16.5)	581 (18.0)	216 (18.0)	253 (18.4)	11 (16.4)	128 (18.3)	228 (19.8)
Women	9568 (81.6)	2162 (80.7)	1098 (83.5)	2648 (82.0)	981 (82.0)	1125 (81.6)	56 (83.6)	573 (81.7)	925 (80.2)
No. of csDMARD, *N* (%)									
≤1	1790 (15.3)	517 (19.3)	133 (10.1)	450 (13.9)	150 (12.5)	205 (14.9)	26 (38.8)	133 (19.0)	176 (15.3)
2	5156 (44.0)	1086 (40.5)	590 (44.9)	1378 (42.7)	597 (49.9)	666 (48.3)	21 (31.3)	337 (48.1)	481 (41.7)
3	3586 (30.6)	798 (29.8)	437 (33.2)	1046 (32.4)	369 (30.8)	372 (27.0)	14 (20.9)	180 (25.7)	370 (32.1)
≥4	1188 (10.1)	279 (10.4)	155 (11.8)	355 (11.0)	81 (6.8)	135 (9.8)	6 (9.0)	51 (7.3)	126 (10.9)
Steroids use, mg/day, median (IQR)	4.5 (2.8–6.2)	4.7 (2.9–6.3)	3.9 (2.5–5.9)	4.7 (2.9–6.5)	4.2 (2.5–6.0)	4.1 (2.6–6.0)	4.3 (3.3–6.4)	3.9 (2.5–6.1)	4.8 (3.2–6.4)
Enrollment year, *N* (%)									
2011	1060 (9.0)	421 (15.7)	161 (12.2)	450 (13.9)	0 (0)	0 (0)	25 (37.3)	0 (0)	3 (0.3)
2012	1381 (11.8)	547 (20.4)	167 (12.7)	641 (19.9)	0 (0)	0 (0)	17 (25.4)	0 (0)	9 (0.8)
2013	1294 (11.0)	466 (17.4)	219 (16.7)	488 (15.1)	68 (5.7)	29 (2.1)	9 (13.4)	0 (0)	15 (1.3)
2014	1822 (15.5)	387 (14.4)	284 (21.6)	386 (12.0)	239 (20.0)	263 (19.1)	2 (3.0)	0 (0)	261 (22.6)
2015	1393 (11.9)	237 (8.8)	165 (12.5)	268 (8.3)	228 (19.0)	246 (17.9)	3 (4.5)	2 (0.3)	244 (21.2)
2016	1388 (11.8)	183 (6.8)	122 (9.3)	351 (10.9)	212 (17.7)	274 (19.9)	4 (6.0)	7 (1.0)	235 (20.4)
2017	1458 (12.4)	181 (6.8)	90 (6.8)	312 (9.7)	232 (19.4)	287 (20.8)	3 (4.5)	171 (24.4)	182 (15.8)
2018	1765 (15.1)	239 (8.9)	101 (7.7)	304 (9.4)	205 (17.1)	245 (17.8)	4 (6.0)	485 (69.2)	182 (15.8)
2019	159 (1.4)	19 (0.7)	6 (0.5)	29 (0.9)	13 (1.1)	34 (2.5)	0 (0)	36 (5.1)	22 (1.9)
CCI, *N* (%)									
1–2	5080 (43.3)	1239 (46.2)	614 (46.7)	1635 (50.6)	481 (40.2)	492 (35.7)	24 (35.8)	244 (34.8)	351 (30.4)
3–4	4125 (35.2)	902 (33.7)	462 (35.1)	1050 (32.5)	429 (35.8)	540 (39.2)	16 (23.9)	275 (39.2)	451 (39.1)
≥5	2515 (21.5)	539 (20.1)	239 (18.2)	544 (16.8)	287 (24.0)	346 (25.1)	27 (40.3)	182 (26.0)	351 (30.4)
FU duration, year, median (IQR)	2.7 (0.8–5.7)	5.4 (1.7–6.7)	4.0 (1.1–6.1)	4.8 (1.1–6.5)	1.4 (0.5–3.1)	1.8 (0.7–3.1)	6.4 (5.3–7.5)	0.6 (0.3–1.0)	1.7 (0.6–3.3)

The number of DMARD types prescribed within 1 year prior to initial biopharmaceutical drug prescription was considered

csDMARDs: methotrexate, sulfasalazine, azathioprine, hydroxychloroquine sulfate, cyclosporine, cyclophosphamide, lefunomide

*bDMARD* biological disease-modifying anti-rheumatic drugs, *tsDMARD* targeted synthetic disease-modifying anti-rheumatic drugs, *csDMARD* conventional synthetic disease-modifying antirheumatic drugs, *TNF* tumor necrosis factor, *N* number of people, *SD* standard deviation, *CCI* Charlson comorbidity index, *FU* follow-up, *IQR* interquartile range

### Overall HZ during initial bDMARD or tsDMARD treatment

Among 11,720 patients with RA and 34,702 person-years of follow-up, 1686 patients (14.4%) experienced HZ during first-line bDMARD or tsDMARD treatment. In the multivariate analysis evaluating the risk of HZ according to the initial bDMARD or tsDMARD type, tofacitinib (aHR, 2.46; 95% CI, 1.61–3.76; *P* < 0.001), infliximab (aHR, 1.36; 95% CI, 1.06–1.74; *P* = 0.017), and adalimumab (aHR, 1.29; 95% CI, 1.02–1.64; *P* = 0.032) were significantly associated with increased HZ risk compared to that of abatacept (Table 2). The median time-to-event was shortest in the tofacitinib group (0.5 years; IQR, 0.2 to 0.9 years).

**Table 2:** Risk of herpes zoster on rheumatoid arthritis patients during first bDMARD or tsDMARD use

	Events	Person-years	Incidence	Time-to-event^a^	aHR (95% CI)	***P*** value	aHR (95% CI)	***P*** value
Etanercept (*n*=2638)	460 (17.2)	10,593	43.4	2.5 (1.0–4.2)	Reference		1.19 (0.94–1.51)	0.146
Infliximab (*n*=1288)	238 (18.1)	4474	53.2	2.1 (0.9–3.7)	1.14 (0.95–1.36)	0.156	1.36 (1.06–1.74)	0.017
Adalimumab (*n*=3176)	526 (16.3)	11,749	44.8	2.1 (0.9–3.7)	1.09 (0.94–1.25)	0.273	1.29 (1.02–1.64)	0.032
Golimumab (*n*=1189)	130 (10.9)	2234	58.2	1.6 (0.8–2.8)	0.89 (0.69–1.14)	0.358	1.06 (0.79–1.42)	0.692
Tocilizumab (*n*=1359)	134 (9.7)	2619	51.2	1.2 (0.5–2.1)	0.74 (0.58–0.94)	0.013	0.88 (0.67–1.16)	0.369
Rituximab (*n*=65)	17 (25.4)	328	51.8	2.6 (1.5–5.0)	1.01 (0.52–1.97)	0.970	1.21 (0.60–2.41)	0.593
Tofacitinib (*n*=687)	48 (6.8)	478	100.4	0.5 (0.2–0.9)	2.06 (1.38–3.08)	<0.001	2.46 (1.61–3.76)	<0.001
Abatacept (*n*=1134)	133 (11.5)	2227	59.7	1.5 (0.6–2.6)	0.84 (0.66–1.06)	0.146	Reference	

Adjusted hazard ratios calculated by multivariate Cox proportional hazards regression after adjustments for age, sex, number of csDMARD, the Charlson comorbidity index, enrollment year, steroids use, and history of zoster

Incidence calculated as the number of events per 1000 person-years

^a^Years to herpes zoster among participants with event, median (interquartile range)

*bDMARD* biological disease-modifying anti-rheumatic drugs, *tsDMARD* targeted synthetic disease-modifying anti-rheumatic drugs, *aHR* adjusted hazard ratio, *CI* confidence interval, *csDMARD* conventional synthetic disease-modifying antirheumatic drugs

Among the adjusted variables, age (HR, 1.02; 95% CI, 1.01–1.02; *P* < 0.001), female sex (HR, 1.18; 95% CI, 1.02–1.37; *P* = 0.032), CCI (HR, 1.05; 95% CI, 1.03–1.07; *P* < 0.001), year of the index date, glucocorticoid use per 1 mg/day increase (HR, 1.02; 95% CI, 1.00–1.04; *P* = 0.033), and history of HZ (HR, 1.54; 95% CI, 1.33–1.78; *P* < 0.001) were found to be independent risk factors for overall HZ risk (Supplementary Table 1). After excluding patients who started bDMARDs or tsDMARD before 2017 to normalize the clinical application period of tofacitinib in South Korea, tofacitinib (aHR, 3.26; 95% CI, 1.63–6.51; *P* < 0.001) showed an increased HZ risk compared to that of abatacept (Supplementary Table 2).

### Incident and recurrent HZ during initial bDMARD or tsDMARD treatment

Among the 9998 patients without a history of HZ and 31,034 person-years of follow-up, 1372 incident HZs (13.7%) occurred (Supplementary Table 3). When analyzing patients without a history of HZ for the evaluation of incident HZ risk, tofacitinib (aHR, 1.99; 95% CI, 1.18–3.37; *P* = 0.011) and infliximab (aHR, 1.43; 95% CI, 1.07–1.91; *P* = 0.015) increased the HZ risk compared to that of abatacept.

In addition, 314 recurrent HZ events (18.2%) were found in 1722 patients with a history of HZ and 3650 person-years of follow-up (Table 3). The duration of previous HZ to the index date was longest and shortest in the golimumab (median, 3.2 years; IQR, 1.5–4.7) and etanercept groups (median, 1.9 years; IQR, 0.8–3.3), respectively. An increased risk of recurrent HZ was found with tofacitinib only in the adjusted analysis (aHR, 3.69; 95% CI, 1.77–7.69; *P* < 0.001) compared to that with abatacept. The incidence was highest and lowest in the tofacitinib (185.6/1,000 person-years) and etanercept groups (70.7/1,000 person-years), respectively, which were much higher than the incidence of incident HZ. Among patients with recurrent HZ who received tofacitinib, HZ occurred in half of the patients at 0.3 years (IQR, 0.1–0.8).

**Table 3:** Risk of recurrent herpes zoster on rheumatoid arthritis patients with history of herpes zoster during first bDMARD or tsDMARD use

	Events	Person-years	Incidence	Previous zoster to index date^a^	Time-to-event^b^	aHR (95% CI)	***P*** value	aHR (95% CI)	***P*** value
Etanercept (*n*=311)	71 (22.8)	1004	70.7	1.9 (0.8–3.3)	1.7 (0.8–3.3)	Reference		1.22 (0.75–2.00)	0.421
Infliximab (*n*=186)	35 (18.8)	435	80.5	2.2 (1.1–3.8)	1.8 (0.4–3.1)	0.88 (0.55–1.41)	0.599	1.08 (0.62–1.87)	0.784
Adalimumab (*n*=412)	96 (23.3)	1018	94.3	2.2 (0.9–3.9)	1.2 (0.5–2.6)	1.22 (0.85–1.75)	0.276	1.50 (0.93–2.40)	0.094
Golimumab (*n*=190)	26 (13.7)	310	83.9	3.2 (1.5–4.7)	0.9 (0.5–1.6)	0.54 (0.27–1.09)	0.084	0.66 (0.32–1.37)	0.268
Tocilizumab (*n*=247)	34 (13.8)	392	86.7	3.0 (1.5–4.8)	0.9 (0.3–1.8)	0.95 (0.57–1.58)	0.838	1.16 (0.67–2.00)	0.590
Tofacitinib (*n*=143)	18 (12.6)	97	185.6	3.9 (2.0–6.2)	0.3 (0.1–0.8)	3.01 (1.49–6.11)	0.002	3.69 (1.77–7.69)	<0.001
Abatacept (*n*=228)	33 (14.5)	394	83.8	2.9 (1.4–4.6)	1.1 (0.5–2.3)	0.82 (0.50–1.34)	0.421	Reference	

Adjusted hazard ratios calculated by multivariate Cox proportional hazards regression after adjustments for age, sex, number of csDMARD, Charlson comorbidity index, enrollment year, steroids use, and the period between the date of previous zoster and the index date

Incidence calculated as the number of events per 1000 person-years

^a^Period between the date of previous herpes zoster to the index date, median (interquartile range)

^b^Years to herpes zoster among participants with event, median (interquartile range)

*bDMARD* biological disease-modifying anti-rheumatic drugs, *tsDMARD* targeted synthetic disease-modifying anti-rheumatic drugs, *aHR* adjusted hazard ratio, *CI* confidence interval, *csDMARD* conventional synthetic disease-modifying antirheumatic drugs

### Subgroup analysis on the association of HZ risk with bDMARDs or tsDMARD during initial bDMARD or tsDMARD treatment

When patients were stratified according to sex, age, total number of DMARDs, CCI, and glucocorticoid use, several subgroups of tofacitinib, infliximab, and adalimumab showed increased HZ risk compared to that of the corresponding abatacept subgroups (Table 4). Additionally, etanercept, infliximab, adalimumab, and tofacitinib were associated with HZ risk in patients with CCI >2. The results of subgroup analyses were generally in accordance with the primary results analyzed from the overall patients, and no significant interaction was found between the factors used for stratification and bDMARDs or tsDMARD in HZ risk.

**Table 4:** Subgroup analysis on risk of herpes zoster on rheumatoid arthritis patients during first bDMARD or tsDMARD use

	Etanercept	Infliximab	Adalimumab	Golimumab	Tocilizumab	Rituximab	Tofacitinib	Abatacept	***P***_**interaction**_
Sex									0.783
Men	1.79 (0.95–3.37)	1.72 (0.85–3.47)	2.13 (1.14–3.99)*	1.52 (0.72–3.25)	1.16 (0.56–2.41)	1.26 (0.16–9.90)	2.51 (0.81–7.83)	Reference	
Women	1.09 (0.84–1.41)	1.26 (0.97–1.66)	1.15 (0.89–1.48)	0.97 (0.71–1.33)	0.84 (0.62–1.13)	1.17 (0.56–2.44)	2.41 (1.52–3.81)***	Reference	
Age, years									0.163
≥65	1.39 (0.90–2.16)	1.21 (0.74–1.99)	1.43 (0.91–2.24)	1.12 (0.65–1.94)	1.06 (0.65–1.73)	0.48 (0.06–3.64)	3.82 (1.92–7.59)***	Reference	
<65	1.13 (0.85–1.51)	1.36 (1.01–1.83)*	1.21 (0.92–1.59)	1.00 (0.71–1.42)	0.79 (0.57–1.10)	1.35 (0.64–2.84)	1.93 (1.12–3.34)*	Reference	
No. of csDMARD									0.993
≤2	1.23 (0.90–1.68)	1.38 (0.98–1.94)	1.34 (0.98–1.83)	1.24 (0.85–1.79)	0.93 (0.65–1.32)	1.48 (0.66–3.30)	2.45 (1.44–4.17)**	Reference	
>2	1.13 (0.79–1.63)	1.34 (0.92–1.95)	1.23 (0.86–1.75)	0.83 (0.52–1.34)	0.82 (0.53–1.29)	0.84 (0.20–3.49)	2.39 (1.16–4.94)*	Reference	
CCI									0.649
≤2	0.77 (0.51–1.16)	1.00 (0.65–1.53)	0.90 (0.60–1.35)	0.77 (0.47–1.26)	0.64 (0.39–1.04)	0.73 (0.22–2.42)	1.52 (0.65–3.54)	Reference	
>2	1.43 (1.07–1.91)*	1.48 (1.08–2.03)*	1.46 (1.10–1.96)*	1.17 (0.82–1.67)	0.99 (0.71–1.38)	1.48 (0.63–3.45)	2.86 (1.75–4.69)***	Reference	
Steroids^a^									0.132
≥5 mg/day	1.20 (0.85–1.68)	1.52 (1.05–2.20)*	1.43 (1.02–1.99)*	1.07 (0.69–1.65)	1.01 (0.68–1.51)	2.50 (1.05–5.95)*	1.92 (1.03–3.59)*	Reference	
<5 mg/day	1.18 (0.84–1.64)	1.22 (0.87–1.73)	1.16 (0.83–1.62)	1.03 (0.70–1.53)	0.78 (0.53–1.14)	0.57 (0.18–1.86)	3.24 (1.80–5.83)***	Reference	

Adjusted hazard ratios calculated by multivariate Cox proportional hazards regression after adjustments for age, sex, hospital type, number of csDMARD, Charlson comorbidity index, enrollment year, and steroids use

^a^Daily dosage within 6 months after the index date

*bDMARD* biological disease-modifying anti-rheumatic drugs, *tsDMARD* targeted synthetic disease-modifying anti-rheumatic drugs, *aHR* adjusted hazard ratio, *CI* confidence interval, *NA* not applicable, *csDMARD* conventional synthetic disease-modifying antirheumatic drugs

*P<0.05. **P<0.01. ***P<0.001

### Event rates and HZ-free survival estimation of bDMARD- or tsDMARD-dependent HZ

**Fig. 2:**
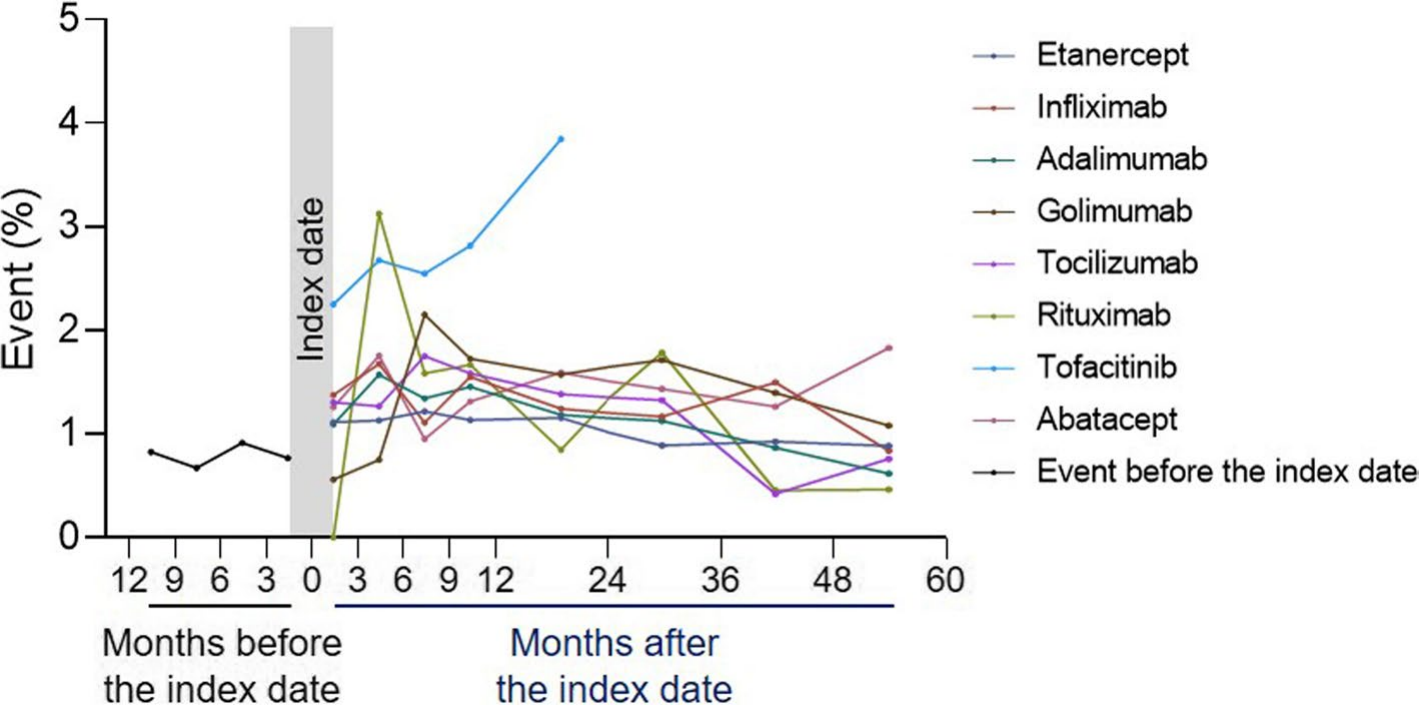
bDMARD-dependent proportion of herpes zoster events in rheumatoid arthritis patients. Periods, including 12–24, 24–36, 36–48, and 48–60 months, were divided by 4 for the normalization of event rates to 3-month periods. Each dot is located in the middle of the month, which represents between-period event rates. The index date refers to the initial date of first-line bDMARD treatment. Patients with censored follow-up investigation were excluded from the calculation of the corresponding event rate

As shown in Fig. 2, most bDMARDs or tsDMARD showed higher HZ event rates compared to that of the event rate before bDMARD or tsDMARD use. The 3-month event rates before the index date varied between 0.7 and 0.9% within 12 months before the index date. Tofacitinib had notably higher event rates (within 3 months, 2.3%; between 3 and 6 months, 2.7%; between 6 and 9 months, 2.5%; between 9 and 12 months, 2.8%; between 12 and 24 months after bDMARD or tsDMARD treatment, 3.8%), of which the event rates were 2.9- to 3.5-fold higher compared to those within 3 months prior to the index date, compared to any other bDMARDs, except for rituximab, which showed an event rate between 3 and 6 months after treatment. Kaplan–Meier estimations of overall, incident, and recurrent HZ-free survival are shown in Supplementary Fig. 2.

### Characteristics of patients who developed HZ

Compared to that of patients who did not develop HZ, those who developed HZ were older and had a higher proportion of women, higher glucocorticoid use, higher proportion with a history of HZ, and longer duration of initial bDMARD or tsDMARD treatment (Supplementary Table 4). When HZ cases were stratified according to the date of the event, very early HZ (≤6 months) was associated with older age, a higher proportion of patients with a history of HZ, and a shorter duration of initial bDMARD or tsDMARD treatment (Supplementary Table 5).

## Discussion

In this analysis of real-world data, seropositive RA patients treated with first-line biologics or tofacitinib were at high HZ risk, and the HZ risk among RA patients with a history of HZ was approximately 1.5-fold higher than that among RA patients without a history of HZ. In addition, the risk of incident and recurrent HZ was significantly increased among tofacitinib users compared to that among users of other biologics. The association was significant even after controlling for potentially confounding factors including age, glucocorticoid use, and comorbidities.

Although no study has explored the risk of recurrent HZ among RA patients with a history of HZ, several studies evaluating the comparative risks of HZ associated with tofacitinib and bDMARDs for RA reported that tofacitinib had a significantly higher HZ risk, whereas other biologics were comparable to each other [13, 14]. Similarly, we found a significant association between tofacitinib and increased HZ risk, especially in RA patients with a history of HZ, which was much higher than the risk of incident HZ among those without a history of HZ. While previous studies reported conflicting data, our study has some novel points compared to previous results in literature, considering strict exclusion of incident HZ cases occurring within 1 month after the first-line bDMARD treatment, comprehensiveness in the evaluation of bDMARD-dependent overall, incident, and recurrent HZ risks, and performance of the sensitivity analysis to support primary findings.

To date, some studies have compared the effects of bDMARDs on the risk of developing HZ. Strangfeld et al. [11] found an increased risk of HZ after treatment with TNFα inhibitors, including infliximab and adalimumab, but not with etanercept, which is in accordance with our results. In addition, a meta-analysis of seven registries revealed that TNFα inhibitors increased HZ risk up to approximately 60%, whereas other studies that were not included in the pooled analysis indicated different results [23, 24]. In contrast, a Japanese health insurance database study demonstrated that both TNFα and non-TNFα inhibitors did not increase HZ risk [12]. According to our data, the risk of overall HZ was increased with infliximab, adalimumab, and tofacitinib, and the incident HZ risk was increased with infliximab and tofacitinib. Considering different study concepts, such as washout periods for HZ and unlimited follow-up for HZ after drug failure, future meta-analyses pooling results of previous studies need to consider study concepts.

Conventionally, a history of viral disease is likely to be considered a protective factor for recurrence, despite a decrease in protective effects over time. A previous study based on a 6.5-year survey reported that prior HZ reduced the incidence of recurring HZ to 31.7% in the HZ-naïve population [25]. However, RA patients are immunocompromised hosts and immunomodulated by various DMARD or glucocorticoid treatments, which may be associated with independently increased HZ. In this study, the incidence of HZ was higher in RA patients with a history of HZ receiving bDMARD or tsDMARD treatment than those in RA patients in the USA and the general population in South Korea [14, 26]. In addition, the incidence rate was relatively higher among RA patients with a history of HZ compared to that of RA patients without a history of HZ (3.35/100 person-years versus 1.6–2.5/100 person-years) [27]. Therefore, RA patients with a history of HZ receiving bDMARDs or tsDMARD treatment may be at higher risk of recurrent HZ compared to those without a history of HZ as found in this study.

Immunity modulated by cells is important in the regulation of the varicella virus, and reduced CD4 T-cell function by the varicella-zoster virus increases HZ risk [28]. As for RA patients, signs of impaired cellular immunity were found, which affected both varicella zoster virus-specific and general effector T cells, and the effects were strongest upon bDMARD treatment [29]. Tofacitinib was found to deteriorate CD4 T-cell proliferation and IFN-γ production in vitro [30]. In addition, innate antiviral defense depends on interferon signaling through the Janus kinase 1 receptor, which is suppressed by tofacitinib [31]. Although the specific mechanisms underlying the contribution of tofacitinib in the development of HZ remain unclear, another Janus kinase inhibitor, ruxolitinib, which is used in patients with myelofibrosis, was also found to increase HZ risk [32]. Therefore, RA patients may be at higher risk for HZ when using tofacitinib in terms of HZ risk, especially in those with a history of HZ.

Some underlying limitations must be considered when interpreting the data. First, the follow-up duration of the tofacitinib group was short due to active prescription after 2017, which may have caused bias in comparison with other bDMARDs. Therefore, we strictly limited the follow-up period to the first bDMARD drug survival, and HZ cases that occurred after drug failure or switching were not counted as events in the present study. Second, some clinical information associated with RA disease activity itself, such as C-reactive protein levels, disease activity score of 28 joints (DAS-28), and Simplified Disease Activity Index (SDAI), were not available; thus, they could not be considered in the present study despite the potential contribution of RA activity to the development of HZ. Future studies with additional clinical information are required to validate the independent association between bDMARD and HZ risk. In addition, the HIRA database may not have captured all HZ cases. However, we believe that most cases may have been identified considering the high accessibility to the hospital and common HZ-related manifestations, such as skin lesions and pain, that require a hospital visit. Another concern is that we could not consider all potential confounders that were found to be associated with the risk of HZ, such as family history of HZ and mitochondrial haplogroups, due to data unavailability [33, 34]. In addition, the adjusted analyses were based on steroid use after the follow-up investigation for HZ regarding the potential synergism between steroids and bDMARDs or tsDMARD. Future studies need to evaluate the potential association between steroid use before initiation of bDMARD or tsDMARD and the risk of HZ. Lastly, information regarding HZ vaccination was unavailable in our database. In South Korea, no HZ vaccines are available, and evidence regarding HZ vaccination in patients with RA is insufficient. In our study population, the HZ vaccination inoculation rate would not be high, and thus, our real-world data consisting of a population with typically no vaccination may provide guidance for the establishment of future recommendations. Despite these limitations, this study is the first to compare bDMARDs in terms of the incidence and recurrence of HZ.

## Conclusions

Patients with seropositive RA treated with bDMARDs or tsDMARD have a high HZ risk. In addition, HZ risk is significantly increased in RA patients with a history of HZ after the initiation of bDMARDs or tsDMARD, unlike that in the general population where prior HZ may reduce the risk of recurrence. RA patients who received tofacitinib showed higher overall, incident, and recurrent HZ risks than those who received other bDMARDs. Individual characteristics, as well as history of HZ, need to be taken into account when initiating bDMARDs or tsDMARD for RA patients to assess future HZ risk.

## Supplementary Information

**Additional file 1: Supplementary Table 1.** Hazard ratios of the covariates adjusted in the analysis on risk of herpes zoster in rheumatoid arthritis patients during first bDMARD or tsDMARD use. **Supplementary Table 2.** Risk of herpes zoster on rheumatoid arthritis patients started bDMARD or tsDMARD from 2017 during first bDMARD or tsDMARD use. **Supplementary Table 3.** Risk of incident herpes zoster on rheumatoid arthritis patients without history of herpes zoster during first bDMARD or tsDMARD use. **Supplementary Figure 1.** Study design. **Supplementary Figure 2.** Kaplan-Meier estimation on risk of herpes zoster among RA patients according to the first-line bDMARDs or tsDMARD. **Supplementary Table 4.** Characteristics of the patients with and without herpes zoster. **Supplementary Table 5.** Characteristics of the patients with and without very early herpes zoster.

## Data Availability

The data that support the findings of this study are available from the HIRA (www.hira.or.kr); however, restrictions apply to the availability of these data, which were used under license for the current study and so are not publicly available.

## Abbreviations

DMARD: Disease-modifying antirheumatic drug
bDMARD: Biologic DMARD
tsDMARD: Targeted synthetic DMARD
HZ: Herpes zoster
TNF: Tumor necrosis factor
HIRA: Health Insurance Review & Assessment Service
CCI: Charlson comorbidity index
aHR: Adjusted hazard ratio
CI: Confidence interval
IQR: Interquartile range
