# Supplementary material for: Incident and recurrent herpes zoster for first-line bDMARD and tsDMARD users in seropositive rheumatoid arthritis patients: a nationwide cohort study
Source: Arthritis Res Ther. 2022 Jul 28;24:180. doi: 10.1186/s13075-022-02871-1 (PMC9330646; doi:10.1186/s13075-022-02871-1)
Supplement: Supplementary file 1 — Additional file 1: Supplementary Table 1. Hazard ratios of the covariates adjusted in the analysis on risk of herpes zoster in rheumatoid arthritis patients during first bDMARD or tsDMARD use. Supplementary Table 2. Risk of herpes zoster on rheumatoid arthritis patients started bDMARD or tsDMARD from 2017 during first bDMARD or tsDMARD use. Supplementary Table 3. Risk of incident herpes zoster on rheumatoid arthritis patients without history of herpes zoster during first bDMARD or tsDMARD use. Supplementary Figure 1. Study design. Supplementary Figure 2. Kaplan-Meier estimation on risk of herpes zoster among RA patients according to the first-line bDMARDs or tsDMARD. Supplementary Table 4. Characteristics of the patients with and without herpes zoster. Supplementary Table 5. Characteristics of the patients with and without very early herpes zoster. [file 13075_2022_2871_MOESM1_ESM.docx]

**Supplementary Table 1. Hazard ratios of the covariates adjusted in the analysis on risk of herpes zoster in rheumatoid arthritis patients during first bDMARD or tsDMARD use**

| **Covariate** | **Reference** | **Variable type** | **HR (95% CI)** | ***P* value** |
| --- | --- | --- | --- | --- |
| Age, years | Continuous | Continuous | 1.02 (1.01-1.02) | <0.001 |
| Sex, female | Male | Categorical | 1.18 (1.02-1.37) | 0.032 |
| Number of csDMARDs | Continuous | Continuous | 1.02 (0.96-1.09) | 0.532 |
| Charlson comorbidity index | Continuous | Continuous | 1.05 (1.03-1.07) | <0.001 |
| Year of the index date |  |  |  | 0.006 |
| 2012 | 2011 | Categorical | 0.98 (0.81-1.20) | 0.867 |
| 2013 | 2011 | Categorical | 1.05 (0.86-1.30) | 0.625 |
| 2014 | 2011 | Categorical | 1.68 (1.35-2.08) | <0.001 |
| 2015 | 2011 | Categorical | 2.10 (1.66-2.65) | <0.001 |
| 2016 | 2011 | Categorical | 1.48 (1.13-1.94) | 0.005 |
| 2017 | 2011 | Categorical | 1.58 (1.17-2.13) | 0.003 |
| 2018 | 2011 | Categorical | 1.37 (0.92-2.04) | 0.127 |
| Glucocorticoid use, per 1 mg/day increase^a^ | Continuous | Continuous | 1.02 (1.00-1.04) | 0.033 |
| History of herpes zoster | No | Categorical | 1.54 (1.33-1.78) | <0.001 |

HR calculated by multivariate Cox proportional hazards regression.

^a^Daily dosage of prednisolone equivalents within 6 months after the index date.

Acronyms: bDMARD, biological disease-modifying anti-rheumatic drugs; tsDMARD, targeted synthetic disease-modifying anti-rheumatic drugs; HR, hazard ratio; CI, confidence interval; csDMARD, conventional synthetic disease-modifying antirheumatic drugs.

**Supplementary Table 2. Risk of herpes zoster on rheumatoid arthritis patients started bDMARD or tsDMARD from 2017 during first bDMARD or tsDMARD use.**

|  | **Events** | **Person-years** | **Incidence** | **Time-to-event^a^** | **aHR (95% CI)** | ***P* value** | **aHR (95% CI)** | ***P* value** |
| --- | --- | --- | --- | --- | --- | --- | --- | --- |
| Etanercept (n=433) | 24 (5.5) | 364 | 65.9 | 0.7 (0.3-1.2) | Reference |  | 1.84 (0.87-3.91) | 0.114 |
| Infliximab (n=193) | 11 (5.6) | 160 | 68.8 | 0.4 (0.3-0.6) | 1.08 (0.48-2.42) | 0.853 | 1.98 (0.82-4.83) | 0.131 |
| Adalimumab (n=634) | 33 (5.1) | 546 | 60.4 | 0.5 (0.3-0.9) | 1.10 (0.60-2.01) | 0.770 | 2.01 (0.98-4.13) | 0.057 |
| Golimumab (n=446) | 17 (3.8) | 413 | 41.2 | 0.6 (0.4-0.9) | 0.63 (0.30-1.33) | 0.225 | 1.15 (0.50-2.67) | 0.743 |
| Tocilizumab (n=558) | 28 (4.9) | 527 | 53.1 | 0.6 (0.3-0.9) | 0.69 (0.37-1.30) | 0.255 | 1.27 (0.61-2.65) | 0.521 |
| Tofacitinib (n=679) | 45 (6.5) | 463 | 97.2 | 0.5 (0.3-0.8) | 1.77 (0.99-3.16) | 0.053 | 3.26 (1.63-6.51) | <0.001 |
| Abatacept (n=378) | 14 (3.6) | 331 | 42.3 | 0.4 (0.2-0.7) | 0.54 (0.26-1.16) | 0.114 | Reference |  |

Adjusted hazard ratios calculated by multivariate Cox proportional hazards regression after adjustments for age, sex, number of csDMARD, the Charlson comorbidity index, enrollment year, steroids use, and history of zoster.

Incidence calculated as the number of events per 1,000 person-years.

^a^Years to herpes zoster among participants with event, median (interquartile range).

Acronyms: bDMARD, biological disease-modifying anti-rheumatic drugs; tsDMARD, targeted synthetic disease-modifying anti-rheumatic drugs; aHR, adjusted hazard ratio; CI, confidence interval; csDMARD, conventional synthetic disease-modifying antirheumatic drugs.

**Supplementary Table 3. Risk of incident herpes zoster on rheumatoid arthritis patients without history of herpes zoster during first bDMARD or tsDMARD use.**

|  | **Events** | **Person-years** | **Incidence** | **Time-to-event^a^** | **aHR (95% CI)** | **P value** | **aHR (95% CI)** | **P value** |
| --- | --- | --- | --- | --- | --- | --- | --- | --- |
| Etanercept (n=2,369) | 389 (16.4) | 9,589 | 40.6 | 2.6 (1.1-4.3) | Reference |  | 1.19 (0.91-1.57) | 0.209 |
| Infliximab (n=1,129) | 203 (18.0) | 4,039 | 50.3 | 2.2 (1.0-3.8) | 1.20 (0.99-1.46) | 0.067 | 1.43 (1.07-1.91) | 0.015 |
| Adalimumab (n=2,817) | 430 (15.3) | 10,731 | 40.1 | 2.2 (1.0-3.8) | 1.06 (0.90-1.24) | 0.477 | 1.26 (0.96-1.66) | 0.093 |
| Golimumab (n=1,007) | 104 (10.3) | 1,924 | 54.1 | 1.8 (0.9-2.9) | 0.97 (0.74-1.27) | 0.818 | 1.16 (0.84-1.60) | 0.385 |
| Tocilizumab (n=1,131) | 100 (8.8) | 2,227 | 44.9 | 1.4 (0.6-2.1) | 0.70 (0.53-0.91) | 0.009 | 0.83 (0.60-1.14) | 0.254 |
| Rituximab (n=62) | 16 (25.8) | 310 | 51.6 | 2.6 (1.4-5.1) | 1.08 (0.53-2.18) | 0.833 | 1.29 (0.61-2.70) | 0.507 |
| Tofacitinib (n=558) | 30 (5.4) | 382 | 78.5 | 0.6 (0.3-0.9) | 1.67 (1.02-2.74) | 0.044 | 1.99 (1.18-3.37) | 0.011 |
| Abatacept (n=925) | 100 (10.8) | 1,832 | 54.6 | 1.5 (0.7-2.6) | 0.84 (0.64-1.10) | 0.209 | Reference |  |

Adjusted hazard ratios calculated by multivariate Cox proportional hazards regression after adjustments for age, sex, number of csDMARD, the Charlson comorbidity index, enrollment year, and steroids use.

Incidence calculated as the number of events per 1,000 person-years.

^a^Years to herpes zoster among participants with event, median (interquartile range).

Acronyms: bDMARD, biological disease-modifying anti-rheumatic drugs; tsDMARD, targeted synthetic disease-modifying anti-rheumatic drugs; aHR, adjusted hazard ratio; CI, confidence interval; csDMARD, conventional synthetic disease-modifying antirheumatic drugs.


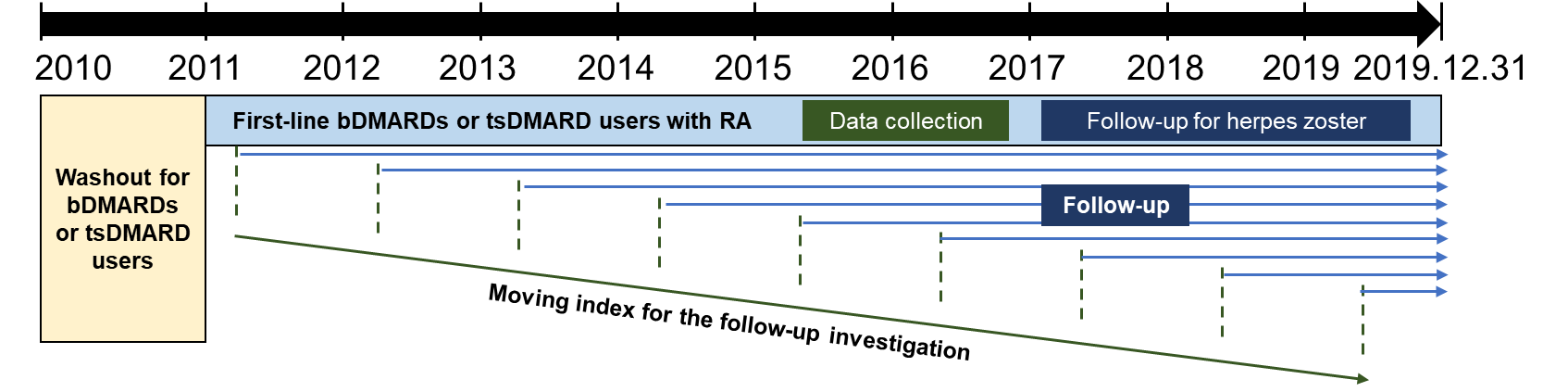


**Supplementary Fig. 1. Study design.** All patients diagnosed with rheumatoid arthritis and prescribed with bDMARDs or tsDMARDs before 2011 were washed out. Data of RA patients with first-line bDMARD or tsDMARDs were obtained in a moving index setting. A follow-up investigation was carried out from the date of bDMARD or tsDMARD prescription to the date of herpes zoster infection, death, or December 31, 2019, whichever occurred earlier during first-line bDMARD or tsDMARD treatment.


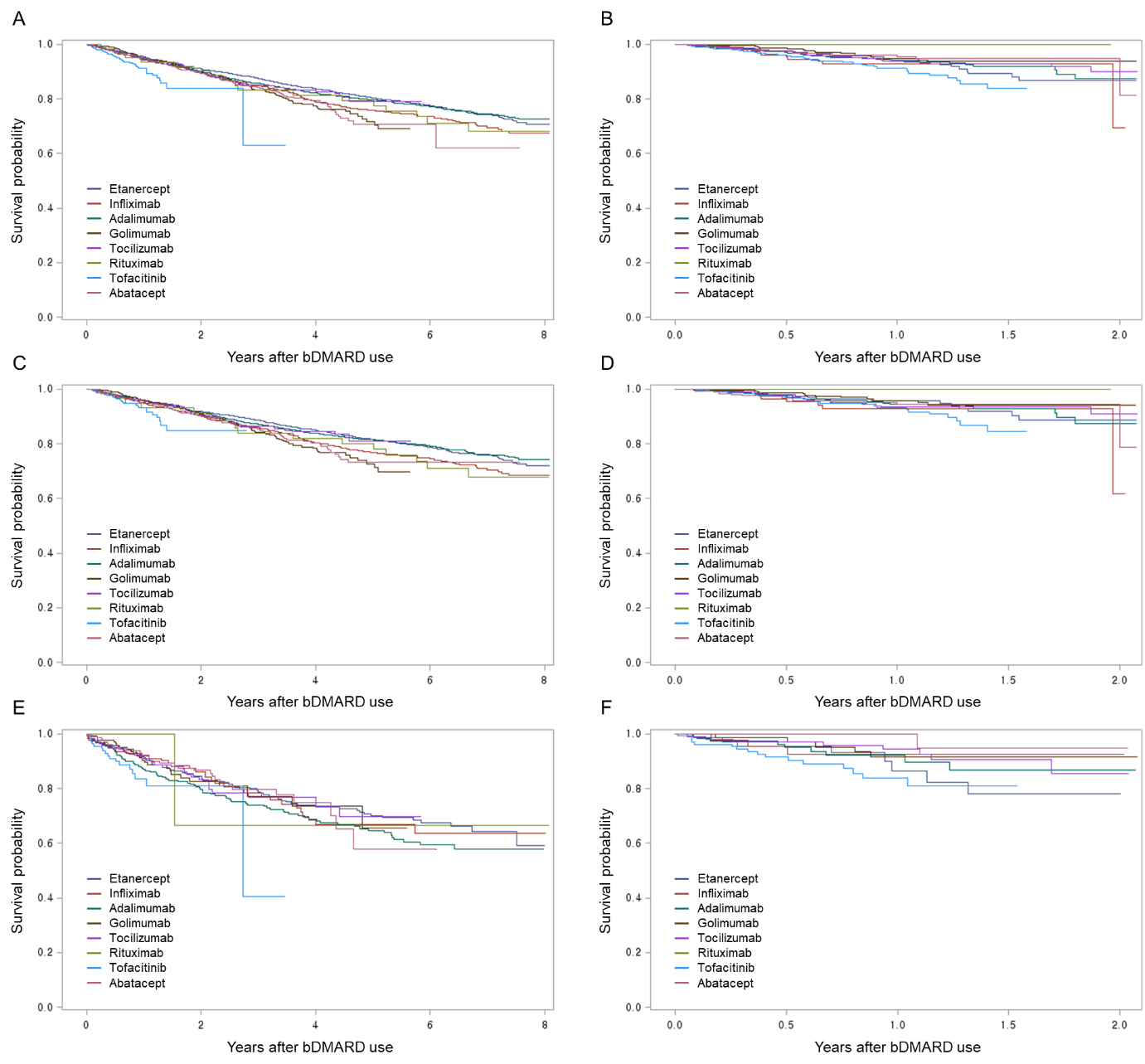


**Supplementary Figure 2. Kaplan-Meier estimation on risk of herpes zoster among RA patients according to the first-line bDMARDs or tsDMARD.** (A) Estimation of overall herpes zoster in overall patients. (B) Estimation of overall herpes zoster in patients after 2017. (C) Estimation of incident herpes zoster in patients without history of herpes zoster. (D) Estimation of incident herpes zoster in patients without history of herpes zoster after 2017. (E) Estimation of overall herpes zoster in patients with history of herpes zoster. (F) Estimation of recurrent herpes zoster in patients with history of herpes zoster after 2017.

**Supplementary Table 4. Characteristics of the patients with and without herpes zoster.**

|  | **Overall participants (n=11,720)** | | |
| --- | --- | --- | --- |
|  | **Herpes zoster**  **(n=1,686)** | **No herpes zoster**  **(n=10,034)** | ***P* value** |
| Age, years | 57 (50-65) | 55 (46-64) | <0.001 |
| Sex, n (%) |  |  | 0.001 |
| Male | 261 (12.1) | 1,891 (87.9) |  |
| Female | 1,425 (14.9) | 8,143 (85.1) |  |
| No. of csDMARD | 2 (2-3) | 2 (2-3) | 0.862 |
| Steroids, mg/day^a^ | 4.7 (2.8-6.5) | 4.4 (2.8-6.2) | 0.038 |
| bDMARD or tsDMARD, n (%) |  |  | <0.001 |
| Etanercept | 460 (17.2) | 2,220 (82.8) |  |
| Infliximab | 238 (18.1) | 1,077 (81.9) |  |
| Adalimumab | 526 (16.3) | 2,703 (83.7) |  |
| Golimumab | 130 (10.9) | 1,067 (89.1) |  |
| Tocilizumab | 134 (9.7) | 1,244 (90.3) |  |
| Rituximab | 17 (25.4) | 50 (74.6) |  |
| Abatacept | 133 (11.5) | 1,020 (88.5) |  |
| Tofacitinib | 48 (6.8) | 653 (93.2) |  |
| Charlson comorbidity index | 3 (2-4) | 3 (2-4) | 0.037 |
| History of herpes zoster, n (%) |  |  | <0.001 |
| Yes | 314 (18.2) | 1,408 (81.8) |  |
| No | 1,372 (13.7) | 8,626 (86.3) |  |
| Follow-up duration, year |  |  |  |
| Herpes zoster-free survival | 1.9 (0.8-3.5) | 2.3 (0.7-5.5) | <0.001 |
| First bDMARD or tsDMARD duration | 5.1 (1.9-6.6) | 2.3 (0.7-5.5) | <0.001 |

Data are median (interquartile range), unless indicated otherwise.

*P* values calculated by t test and chi-square test for continuous and categorical variables, respectively.

^a^Daily dosage within 6 months after the index date.

Acronyms: csDMARD, conventional synthetic disease-modifying antirheumatic drugs; bDMARD, biological disease-modifying anti-rheumatic drugs; tsDMARD, targeted synthetic disease-modifying anti-rheumatic drugs.

**Supplementary Table 5. Characteristics of the patients with and without very early herpes zoster.**

|  | **Very early herpes zoster,**  **≤6 months (n=267)** | **Other herpes zoster,**  **>6 months (n=1,419)** | ***P* value** |
| --- | --- | --- | --- |
| Age, years | 59 (51-67) | 57 (50-64) | 0.012 |
| Sex, n (%) |  |  | 0.902 |
| Male | 42 (16.1) | 219 (83.9) |  |
| Female | 225 (15.8) | 1,200 (84.2) |  |
| No. of csDMARD | 2 (2-3) | 2 (2-3) | 0.003 |
| Glucocorticoid use, mg/day^a^ | 4.7 (2.7-6.3) | 4.7 (2.8-6.5) | 0.712 |
| bDMARD or tsDMARD, n (%) |  |  | <0.001 |
| Etanercept | 55 (12.0) | 405 (88.0) |  |
| Infliximab | 36 (15.1) | 202 (84.9) |  |
| Adalimumab | 77 (14.6) | 449 (85.4) |  |
| Golimumab | 13 (10.0) | 117 (90.0) |  |
| Tocilizumab | 30 (22.4) | 104 (77.6) |  |
| Rituximab | 2 (11.8) | 15 (88.2) |  |
| Tofacitinib | 25 (52.1) | 23 (47.9) |  |
| Abatacept | 29 (21.8) | 104 (78.2) |  |
| Charlson comorbidity index | 3 (2-4) | 3 (2-4) | 0.996 |
| History of herpes zoster, n (%) |  |  | <0.001 |
| Yes | 83 (26.4) | 231 (73.6) |  |
| No | 184 (13.4) | 1,188 (86.6) |  |
| Follow-up duration, year |  |  |  |
| Herpes zoster-free survival | 0.3 (0.2-0.4) | 2.3 (1.3-3.8) | <0.001 |
| First bDMARD or tsDMARD duration | 2.4 (0.8-5.5) | 5.3 (2.4-6.7) | <0.001 |

Data are median (interquartile range), unless indicated otherwise.

*P* values calculated by t test and chi-square test for continuous and categorical variables, respectively.

^a^Daily dosage of prednisolone equivalents within 6 months after the index date.

Acronyms: csDMARD, conventional synthetic disease-modifying antirheumatic drugs; bDMARD, biological disease-modifying anti-rheumatic drugs; tsDMARD, targeted synthetic disease-modifying anti-rheumatic drugs.
